# Accurately Determining the Extent of Coupling in Post Polymerization Reactions of Polystyrene

**DOI:** 10.3390/polym10010080

**Published:** 2018-01-16

**Authors:** Ching Pan, Eric Tillman

**Affiliations:** Department of Chemistry and Biochemistry, Santa Clara University, 500 El Camino Real, Santa Clara, CA 95053, USA; cpan@scu.edu

**Keywords:** ATRP, gel permeation chromatography, polystyrene

## Abstract

Polymers prepared by controlled radical polymerization (CRP) can be employed in subsequent chain-end joining reactions, yet accurately assessing the extent of coupling in mechanistically unique paths is not straightforward. Precisely known mixtures of polystyrene standards were prepared and analyzed by gel permeation chromatography (GPC), mimicking the coupled product and precursor that could be present after a post-polymerization, chain-end joining reaction. The exactly known percentages of each polymer in the mixture allowed for comparison of the true “extent of coupling” (*X*_c_) to that determined by a commonly used equation, which is based on number average molecular weights (*M*_n_) of the precursor and coupled product. The results indicated that an improvement in accuracy could be achieved by instead using refractive index (RI) signal height ratios under the peak molecular weight (*M*_p_) of each component, with all calculations being within 0.05 of the true *X*_c_ of the fabricated “product” mixture (compared to greater than 0.10 average error using the more established method) when the sample mixture had nominal molecular weights of 2500 and 5000 Da. Moreover, when “precursor” and “coupled” pairs mixed were not related as a simple doubling of molecular weight, the calculation method presented here remained effective at determining the content of the mixture, especially at higher *X*_c_ values (>0.45). This second case is important for experiments that may link polymer chains together with a spacer, such as a radical trap, a triazole, or even larger structure such as an oligomer.

## 1. Introduction

**Scheme 1:**
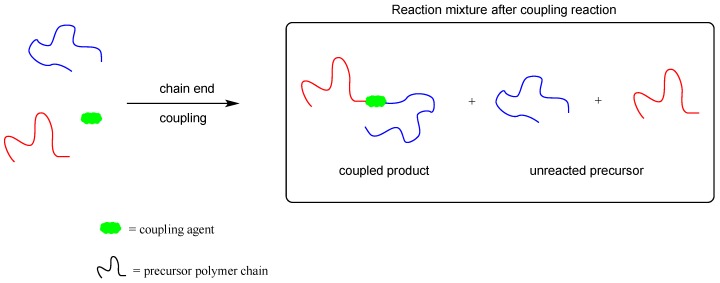
General reaction for linking polymer chains together, which may include the incorporation of a “spacer” between the chains due to the use of a coupling agent. Red and blue lines represent generic polymer chains that may or may not be compositionally equivalent.

As synthetic routes leading to complex macromolecular architectures continue to be a major area of current research that will continue into the foreseeable future [1,2,3,4], polymer chain-end coupling reactions have become an important post-polymerization reaction [5,6,7,8,9,10,11,12]. These can be as simple as two polymer radical chain ends terminating by radical-radical coupling [10,12], but may also involve the insertion of a “spacer” between the polymer chains in the form of a radical trap [5], a triazole ring [7], an oligomeric polymer chain [13], or another functionality [14] (Scheme 1). The ability to accurately assess the extent of coupling (*X*_c_) is vital for the translation of a model coupling reaction into a more complex synthetic sequence, such as using an analogous reaction in an intramolecular cyclization reaction [15].

Synthetic polymer chemists most commonly rely on GPC-RI data to quickly and accurately characterize their polymer products, be it the initial product of the polymerization [16,17,18] or the results of a post-polymerization reaction [19,20]. Determining *X*_c_ in a chain-end joining reaction has been most commonly done using the following equation [9]:

(1) $$X_{c}=2\left(1-\frac{M_{n}\left(precursor\right)}{M_{n}\left(product\right)}\right)$$

that easily allows the experimenter to determine the success of a coupling reaction after obtaining the number averaged molecular weights (*M*_n_) of both the precursor and coupled product. The assumption is that the coupled product does not have any new functionality that affects its hydrodynamic volume, which may not always be the case.

In this contribution, we introduce an analytical method (Equation (2)) to find *X*_c_ that instead uses RI peak height ratios of coupled products compared to unreacted precursors. These results are compared to both the true “*X*_c_” values of the solutions, and the value obtained from Equation (1). The method introduced in this contribution, Equation (2), seeks to eliminate the problem faced when involving the *M*_n_ value of the original precursor in the *X*_c_ equation, because the precursor’s size is not an appropriate comparison due to new mid-chain functionality in the coupled product. Also, there is often no way for the experimenter to know the true value of the completely coupled dimer due to the nature of the spacer, preventing the modification of Equation 1 to better reflect the true *X*_c_. To assess the accuracy of different equations, we use precisely known mixtures of polystyrene and compare the outcome of the analyses to the true “*X*_c_” of the prepared mixtures.

(2) $$X_{c}=\left(\frac{\mathrm{RI}\;\mathrm{peak}\;\mathrm{intensity}\left(product\right)}{\mathrm{RI}\;\mathrm{peak}\;\mathrm{intensity}\left(product\right)+\mathrm{RI}\;\mathrm{peak}\;\mathrm{intensity}\left(precursor\right)}\right)$$

## 2. Materials and Methods

### 2.1. Materials

Copper(I) bromide (CuBr, 98%, Aldrich, St. Louis, MO, USA) and copper metal (Cu^0^, fine powder, Baker & Adamson Easton, PA, USA were used as received. Tetrahydrofuran (THF, inhibitor free ≥99.9%, Aldrich) was obtained from the Aldrich Pure-Pac^TM^ System. Styrene (S, ≥99%, Aldrich) was purified through pre-packed column inhibitor removers (Aldrich). The following were used as received and stored in the refrigerator: 1-bromoethylbenzene (BEB, 97%), and *N*,*N*,*N′*,*N″*,*N″*-pentamethyldiethylenetriamine (PMDETA, 99%, Aldrich). The *N*-*tert*-Butyl-α-phenylnitrone (tBuPN, Aldrich) was used as received and stored in the freezer.

### 2.2. Procedure for the Synthesis of Monobrominated Polystyrene Using Atom Transfer Radical Polymerization (ATRP)

Styrene (4.0 mL, 34.9 mmol), CuBr (100 mg, 0.698 mmol), and (1-bromoethyl)benzene (BEB, 95 μL, 0.698 mmols) were added into a custom-made Schlenk flask, capped with a rubber septum, that was attached to the Schlenk line. The contents of the flask were exposed to three freeze–pump–thawing cycles before being placed on heat plate with custom fit block heaters and equipped with a digital contact thermoregulator (Chemglass CG-1994-V015, Vineland, NJ, USA) set to 80 °C and left to stir for 5 min. PMDETA (146 μL, 0.698 mmol) was then added to the reaction flask via syringe to begin polymerization. The polymerization is stopped after 120 min by exposing the content of the flask to the atmosphere and placing the flask in an ice bath. The reaction mixture is filtered into a GPC vial, diluted with THF, and analyzed.

### 2.3. Typical Procedure for ATRC of PSBr

A representative ATRC reaction was performed as follows: molar ratio of [PSBr]:[CuBr]:[Cu^0^]:[PMDETA] = 1:5:5:10, monobrominated polystyrene (PSBr, 195 mg, 0.075 mmol, 2600 g/mol), CuBr (53.8 mg, 0.375 mmol), Cu^0^ (16 mg, 0.375 mmol), and THF (5 mL) were added into a custom-made Schlenk line round-bottom flask. Concentrations of PSBr were consistently kept at 15 mM. The reaction flask, sealed with a rubber septum, was attached to the Schlenk line. The flask was exposed to three freeze–pump–thawing cycles before being placed onto a heat plate set to 60 °C. After 5 min of heating and stirring, PMDETA (157 μL, 0.75 mmol) was added into the reaction chamber via syringe. The RTA-ATRC reaction is stopped after 60 min by exposing the content of the flask to the atmosphere and placing the flask in an ice bath. The reaction mixture is filtered into a GPC vial, diluted with THF, and analyzed.

### 2.4. Typical Procedure for RTA-ATRC of PSBr

A representative RTA-ATRC reaction was performed as follows: molar ratio of [PSBr]:[CuBr]:[Cu^0^]:[PMDETA] = 1:2.5:2.5:5, monobrominated polystyrene (PSBr, 195 mg, 0.075 mmol, 2600 g/mol), CuBr (26.9 mg, 0.1875 mmol), Cu^0^ (8.2 mg, 0.1875 mmol), *N*-*tert*-Butyl-α-phenylnitrone (tBuPN, 13 mg, 0.075 mmol), and THF (5 mL) were added into a custom-made Schlenk line round-bottom flask. Concentrations of PSBr were consistently kept at 15 mM. The reaction flask, sealed with a rubber septum, was attached to the Schlenk line. The flask was exposed to three freeze–pump–thawing cycles before being placed onto a heat plate set to 80 °C. After 5 min of heating and stirring, PMDETA (78 μL, 0.375 mmol) was added into the reaction chamber via syringe. The RTA-ATRC reaction is stopped after 20 min by exposing the content of the flask to the atmosphere and placing the flask in an ice bath. The reaction mixture is filtered into a GPC vial, diluted with THF, and analyzed.

### 2.5. Typical Procedure to Mimic Various % Coupling Reactions

The amount of PS Standards needed to mimic various percent coupling were calculated and weighed out on to VWR Weighing Paper (Radnor, PA, USA) using a balance scale (Mettler Toledo NewClassic MF Model ML204, Columbus, OH, USA). The measured PS standards were transferred into 25 mL volumetric flasks and 25 mL THF was added into the flask. The solution was mixed well by swirling and inverting the flask. Approximately 2 mL of each solution containing the PS Standards was transferred to a GPC vial for analysis.

### 2.6. Characterization

GPC analysis was done on system comprised of a Shimadzu CBM-20A communications module (Kyoto, Japan), a Shimadzu DGU-20A degassing unit, a Shimadzu SIL-20A auto sampler, a Shimadzu LC-20AD pump, and Wyatt T-rEX RI detector (Santa Barbara, CA, USA). The instrument was interfaced with a PC and was operated using PSS WinGPC Unichrom software (Mainz, Germany). Separations were performed using aPSS SDV analytical 1000 angstrom and a 100,000 angstrom column in sequence, housed inside a Shimadzu CTO-2A column oven set to 40 °C. THF was used as an eluent at an optimized flow rate of 1.00 mL/min, and a 10-point calibration was performed using Agilent EasiCal PS standards (Santa Clara, CA, USA). The data analysis was performed using the PSS WinGPC software.

## 3. Results

**Scheme 2:**
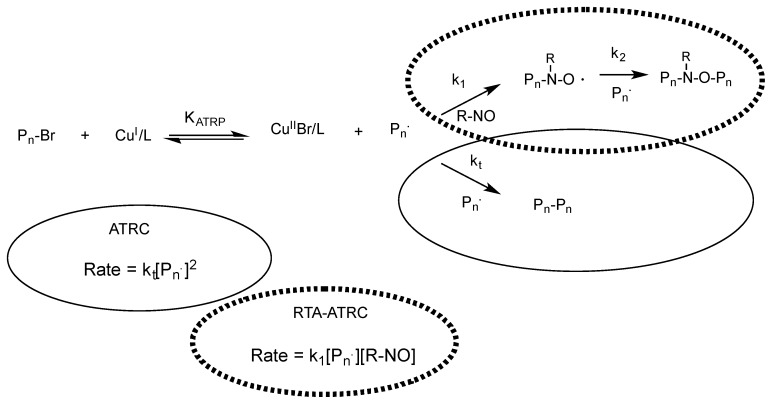
Comparison of the mechanism and rate equation for ATRC (solid circle) and RTA-ATRC. (dashed circle).

ATRP creates polymers with halogen chain ends [21,22,23,24], ready to be employed in post-polymerization coupling reactions such as atom transfer radical coupling (ATRC) [25]. Recently, more environmentally benign analogs have become commonplace among synthetic polymer chemists, such as activators regenerated by electron transfer (ARGET) [26] and electrochemical ATRP (eATRP) [27]. Each of these methods allows the experimenter the advantage of using lower catalyst-ligand loadings without sacrificing control over the polymerization or yield of the polymer. A more robust variation of ATRC uses radical traps to assist in the coupling process in a kinetically and mechanistically distinct sequence named radical trap-assisted ATRC (RTA-ATRC) [28,29,30]. RTA-ATRC, for example, often uses less than half of the catalyst-ligand equivalents compared to traditional ATRC. These post-polymerization processes are compared in Scheme 2.

**Figure 1:**
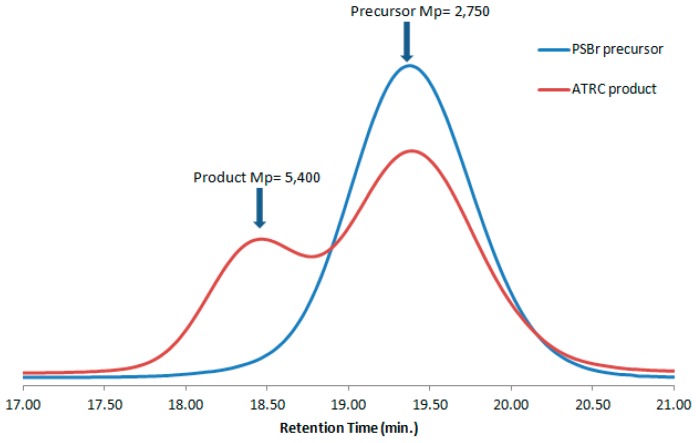

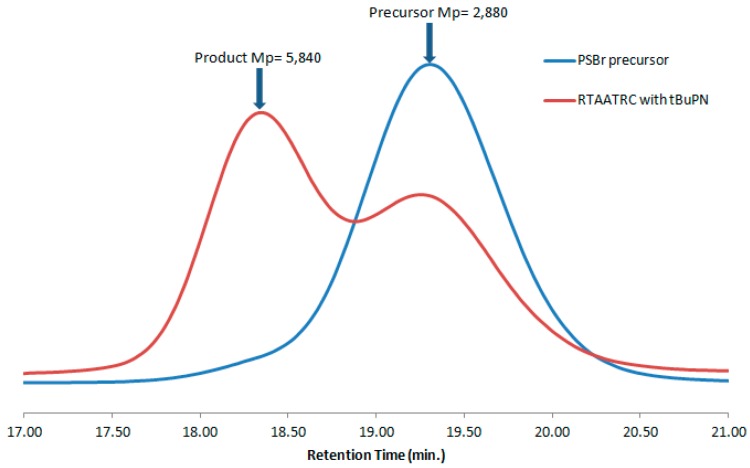
(**Top**) GPC traces of PSBr precursor (blue) and product of an ATRC reaction (red). (**Bottom**) GPC traces of PSBr precursor (blue) and product of RTA-ATRC reaction using *N*-*tert*-Butyl-α-phenylnitrone as the radical trap.

The alkoxyamine spacer incorporated into the polymer (top, dotted circle) means the coupled polymer will not be related as simply twice the size of the precursor. Although we are using RTA-ATRC to illustrate this dilemma, it is broadly applicable to joining polymer chains by click chemistry or any other method that uses a coupling partner. Brominated polystyrene (PSBr) was prepared by ATRP, and was subjected to partial coupling to mimic results obtained in a chain-end joining reaction. It should be noted that both ATRC and RTA-ATRC are capable of near quantitative dimerization, but for the purposes of assessing *X*_c_ we purposely wanted unreacted precursor to remain. As shown in Figure 1 (top), the peak molecular weight (*M*_p_) of the ATRC product is nearly exactly double that of the PSBr precursor from which it was derived. However, when RTA-ATRC is used with *N*-*tert*-Butyl-α-phenylnitrone as the radical trap, the mid-chain alkoxyamine functionality contributes to the hydrodynamic volume of the coupled product and a more than doubling of the *M*_p_ is seen when compared to the precursor. Using Equation (1) is valid for traditional ATRC, but does not take into account any new size imparted into the coupled product as a result of the coupling mechanism.

This submission deals specifically with polystyrene (PS) chains, as they are commonly used in coupling reactions and have readily available standards and calibrants for GPC analysis. By using monomodal standards, precisely known mixtures could be prepared to mimic various “percent coupling”, or *X*_c_, scenarios, where a portion of higher molecular weight, “coupled” product is present in a mixture with lower molecular weight “precursor”. This can be envisioned in Scheme 1, where it requires 2 molar equivalents of “precursor” to produce 1 equivalent of “coupled product”. In the scenario shown, 6 molar equivalents of precursor would partially couple to form 2 molar equivalents of product, meaning 4 of the 6 precursor chains underwent coupling (mimicking *X*_c_ = 0.67). The true *X*_c_ of the simulated reaction mixture containing both “precursor” and “coupled product” is shown in Equation (3):

(3) $$\mathrm{True}\;X_{c}=\left(\frac{2\left(“coupled\;product”\;\mathrm{mmol}\right)}{\left(”precursor”\;\mathrm{mmol}+2(“coupled\;product”\;\mathrm{mmol})\right)}\right)$$

**Figure 2:**
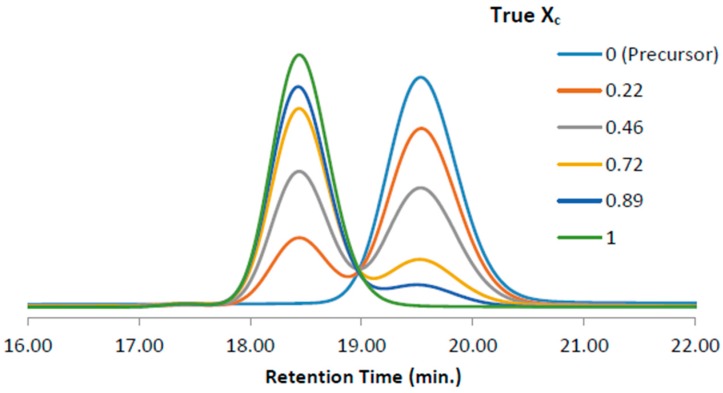
GPC traces of PS standards mixed to mimic varying extents of coupling, as listed in Table 1.

As shown in Table 1, several mixtures of two PS chains (related by the larger being nearly double molecular weight) were prepared and the true “extent of coupling” (*X*_c_) was determined by Equation (3). The solutions were then analyzed by GPC-RI (Figure 2), and the *X*_c_ was calculated using both Equations (1) and (2). We introduce Equation (2) in this submission as a means of computing the extent of coupling in chain-end joining reactions, by simply comparing the relative intensities of the Mp signals attributed to the precursor and coupled peak.

**Figure 3:**
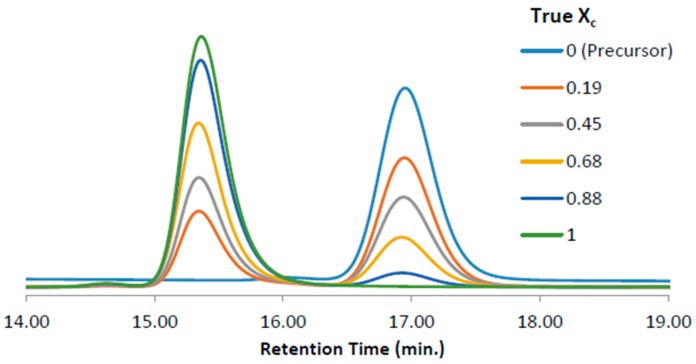
GPC traces of PS standards mixed to mimic varying extents of coupling, as listed in Table 2.

Another pair of PS chains was mixed and the ratios were precisely determined, but in this case the longer chain mimicked the presence of a relatively large “spacer” in the coupled product. Because the “precursor” and “product” would no longer be related as a near doubling of molecular weights, Equation (1) would not be expected to yield numbers that matched the true *X*_c_ values. Conversely, Equation (2) should not be impacted by the fact that the “coupled” product is substantially more than two times the molecular weight of the precursor. Listed in Table 2 are the true *X*_c_ values along with those calculated from GPC data using Equations (1) and (2). In Figure 3, the GPC traces of the varying fractions are shown.

## 4. Discussion

When “precursors” and “coupled product” were nearly related as a doubling in size (Table 1 and Figure 2), both Equations (1) and (2) gave computed *X*_c_ values that were relatively accurate. Perhaps surprisingly, Equation (2) overall yielded more accurate *X*_c_ values, on average being within 0.05 of the true extent of coupling determined by Equation (3). Conversely, values calculated using Equation (1) deviated from true values especially as the extent of coupling increased, and on average deviated by greater than 0.10 of the true *X*_c_ value. This is a situation that would mimic chain-end joining by ATRC or another means that did not introduce a “spacer” that modified the hydrodynamic radius of the chain.

Conversely, Equations (1) and (2) yielded drastically different *X*_c_ values when the “product” was much larger than the “precursor”. In the case of Equation (1), the average difference is greater than 0.355 from the true *X*_c_, while Equation (2) deviates on average by around 0.108. As expected, Equation (1) overestimates the coupling in each case, as the *M*_n_ of the “coupled” product is higher than twice that of the precursor. This is particularly apparent at 0.68 and 0.88 true *X*_c_ values, where calculated values from Equation (1) are greater than are theoretically possible. At extents of coupling that would be more applicable to true synthetic results (0.45 and higher), Equation (2) yields values close to true *X*_c_ values (averaging a deviation of 0.083). This situation would represent chain end joining by where the coupling agent was quite large (a diradical oligomer, for example), and using the precursor’s *M*_n_ in the equation to determine *X*_c_ leads to greater error.

**Figure 4:**
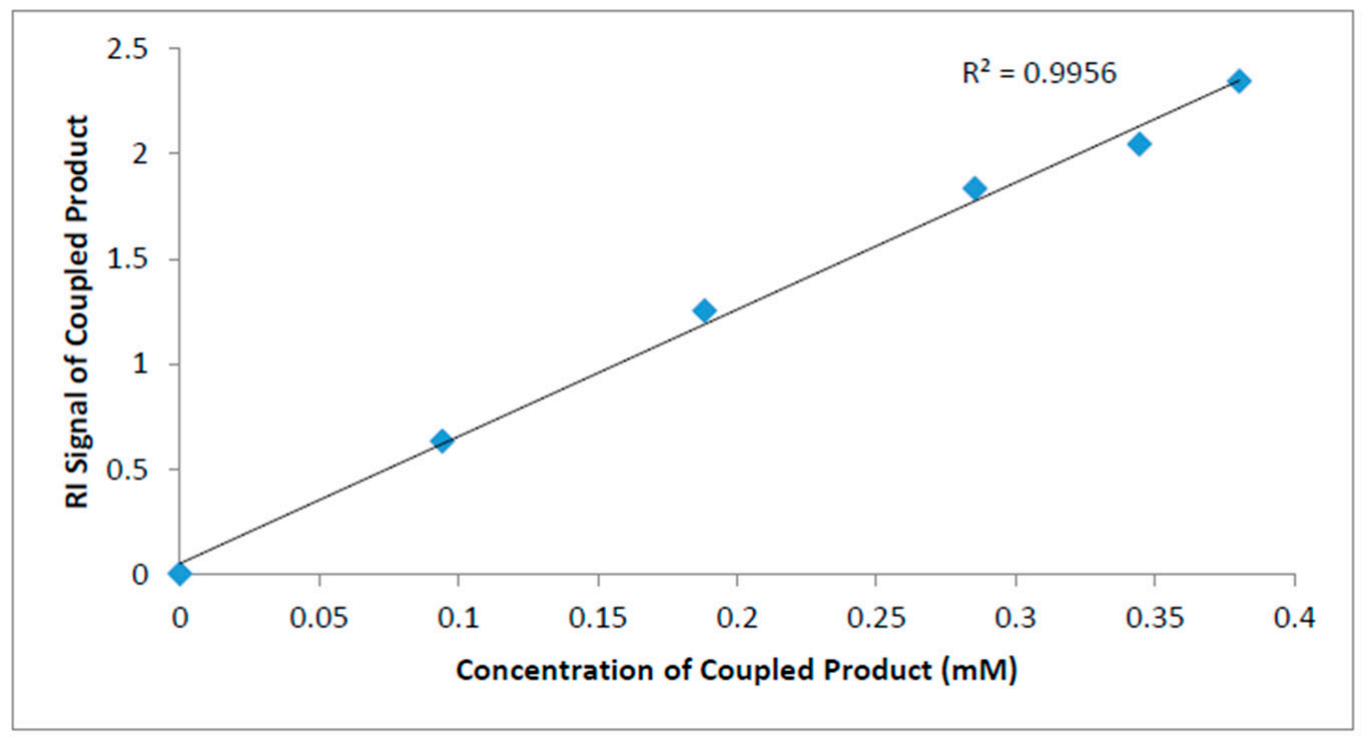
RI signal of GPC-RI detector (Wyatt T-rEX) of *M*_p_ assigned to “coupled” peak of polymer mixture (nominal molecular weights 2500 and 5000 Da), as a function of concentration in the mixture injected into GPC.

**Figure 5:**
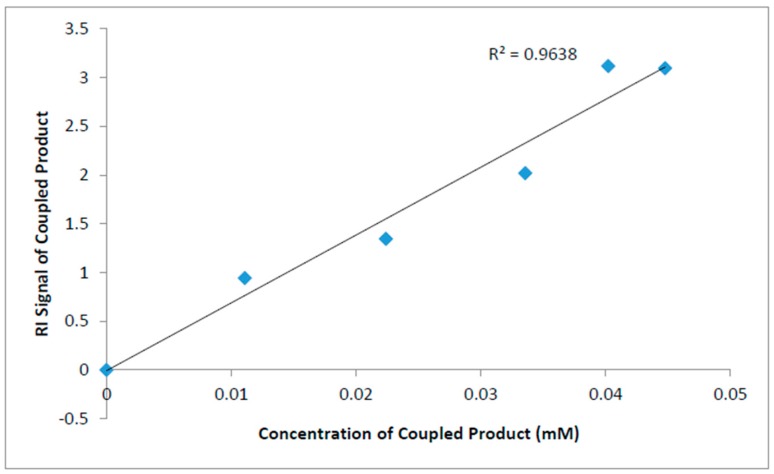
RI signal of GPC-RI detector (Wyatt T-rEX) of *M*_p_ assigned to “coupled” peak of polymer mixture (nominal molecular weights 17,500 and 50,000 Da), as a function of concentration in the mixture injected into GPC.

The refractive index increment (*d*_n_/*d*_c_) values of PS and other polymers show molecular weight sensitivity [31], although these changes in RI signal may be inconsequential compared to built-in errors in most routine GPC analyses. Plotting the RI signal vs. the concentration of the “coupled” product in each mixture results in a near linear relationship, as can be seen in Figure 4 and Figure 5. The concentration of the “coupled product” is comparatively low, as even complete “coupling” would result in a polymer dimer in the reaction mixture that is only half as concentrated as the precursor was at reaction time = 0.

## 5. Conclusions

To conclude, a simple equation relying only on GPC-RI data allows the synthetic polymer chemist to quickly and easily calculate the extent of coupling (*X*_c_) in a reaction mixture containing both coupled product and precursor. The experimentalist does not need to know the true molecular weight of the dimer, but only compare peak height ratios based on easily attainable RI data. The utility of this equation is particularly apparent in situations where a mid-chain spacer is incorporated between the chains in the coupled product, and even more so when a spacer’s apparent size on the GPC instrument is unknown. An advantage of this equation compared to commonly used methods is that the number average molecular weight of the initial precursor used in the coupling sequence is not considered in determining *X*_c_.

## Figures and Tables

**Table 1:** Percent “Coupling” Calculations of Mixed PS Standards 2500:5000 ^a^.

True *X*_c_^b^	*M*_n_^c^	Calculated *X*_c_, Previously Reported ^d^	Error in Equation (1) ^e^	Calculated *X*_c_, Method Reported Here ^f^	Error in Equation (2) ^g^
0	2293	--	--	--	--
0.22	2649	0.27	0.05	0.28	0.01
0.46	3160	0.55	0.09	0.53	0.07
0.72	4086	0.88	0.16	0.81	0.09
0.89	4678	1.02	0.13	0.91	0.02
1.0	5227	--	--	--	--

^a^ Purchased as PS standards from Aldrich, with “nominal molecular weights” listed as 2500 and 5000 Da; ^b^ precisely known value based on the amount of each polymer in the THF solution. See Equation (3) for calculation; ^c^ number average molecular weight based on PS standards using a 10-point calibration curve; ^d^ see Equation (1); ^e^ average error is 0.108; ^f^ see Equation (2); ^g^ average error is 0.048.

**Table 2:** Percent “Coupling” Calculations of Mixed PS Standards 17,500:50,000 ^a^.

True *X*_c_^b^	*M*_n_^c^	Calculated *X*_c_, Previously Reported ^d^	Error in Equation (1) ^e^	Calculated *X*_c_, Method Reported Here ^f^	Error in Equation (2) ^g^
0	15,876	--	--	--	--
0.19	20,859	0.48	0.29	0.37	0.18
0.45	25,079	0.73	0.28	0.55	0.10
0.68	34,297	1.07	0.39	0.77	0.09
0.88	47,790	1.34	0.46	0.94	0.06
1.0	55,917	--	--	--	--

^a^ Purchased as PS standards from Aldrich, with “nominal molecular weights” listed as 17,500 and 50,000 Da; ^b^ precisely known value based on the amount of each polymer in the THF solution using Equation (3); ^c^ number average molecular weight based on PS standards using a 10-point calibration curve; ^d^ see Equation (1); ^e^ average error is 0.355; ^f^ see Equation (2); ^g^ average error is 0.1075.
